# Presepsin production in monocyte/macrophage-mediated phagocytosis of neutrophil extracellular traps

**DOI:** 10.1038/s41598-022-09926-y

**Published:** 2022-04-08

**Authors:** Akishige Ikegame, Akihiro Kondo, Ken Kitaguchi, Kanami Sasa, Masashi Miyoshi

**Affiliations:** 1 Department of Medical Technology, Faculty of Health Sciences, Kagawa Prefectural University of Health Sciences, Takamatsu, Kagawa 761-0123 Japan; 2 Department of Medical Technology, Tokushima University Hospital, Tokushima, Japan

**Keywords:** Cell biology, Biomarkers

## Abstract

Presepsin, a biomarker discovered in Japan, has been clinically applied as a diagnostic aid for sepsis. Recently, however, it has been reported that presepsin levels are elevated in patients with severe systemic lupus erythematosus without infection, suggesting the existence of a production mechanism that does not involve bacterial phagocytosis. In this study, we aimed to elucidate the mechanism of presepsin production without bacterial phagocytosis and explore the clinical significance of presepsin. Neutrophil extracellular traps (NETs) were induced by *Escherichia coli* and phorbol myristate acetate (PMA) in neutrophils isolated from the peripheral blood of healthy subjects. NET induction alone did not increase presepsin levels, but co-culturing with monocytes significantly increased them. The addition of a NET formation inhibitor also suppressed presepsin levels, suggesting that presepsin production is greatly influenced by monocyte phagocytosis of NETs. Phagocytosis of NETs by THP-1 and U937 cells, which was induced by CD14 expression, also increased presepsin levels. This study suggests that presepsin can be used to assess the severity of inflammatory diseases, such as autoimmune diseases, and monitor treatment effects.

## Introduction

Sepsis is a common condition associated with unacceptably high mortality and long-term morbidity in many survivors^1,2^. Therefore, early diagnosis is important for treating sepsis, and because it has been reported to have a significant impact on the subsequent course of the disease, biomarkers that can be measured rapidly with high specificity are required^3,4^.

Cluster of differentiation 14 (CD14) has been described as a monocyte/macrophage differentiation antigen on the surface of myeloid lineages, such as monocytes, macrophages, and dendritic cells (DCs)^5^. This protein plays a crucial role in immune recognition and reactivation of microbial cell wall components in gram-positive and gram-negative bacteria^6^. Recently, CD14 has been shown to play a role in the phagocytic clearance of apoptotic cells^7^. CD14 isoforms (52–55 kDa) expressed on the surfaces of monocytes and neutrophils, membrane protein (mCD14), are attached to the cell surface by a glycosyl phosphatidylinositol (GPI) anchor, while the other isoform is a serum soluble (48–56 kDa) (sCD14, an acute-phase protein)^8^. Soluble CD14 found in human serum has been attributed to the shedding of mCD14 from monocytes, macrophages, and PMNs. Membrane CD14, a receptor for lipopolysaccharide (LPS) on the membrane of mononuclear phagocytes (MPS), binds to LPS-binding protein (LBP) in plasma and transfers it to the cell surface receptor CD14^9^. LPS stimulates human monocyte activation via several intracellular signaling pathways that involve proinflammatory factors^10^.

sCD14 plays an important role in mediating immune responses to LPS in CD14-negative endothelial and epithelial cells. During inflammatory stress, sCD14 is cleaved in the plasma to generate a 13 kDa-N-terminal fragment which has been identified as an sCD14 subtype (sCD14-ST; also known as presepsin)^11–13^.

The mechanism of presepsin production is as follows: when phagocytes phagocytose bacteria infecting blood vessels during sepsis, CD14 expressed on the surface of phagocytes is taken up together with the bacteria and is degraded by neutrophil elastase, a proteolytic enzyme, to produce presepsin^14^. Therefore, presepsin has a high specificity as a sepsis biomarker. Furthermore, presepsin is a useful biomarker for the early detection of sepsis owing to its short half-life (4 h)^15–17^.

On the other hand, elevated presepsin levels have been reported in patients with systemic lupus erythematosus (SLE) and hemophagocytic syndrome, autoimmune diseases without evidence of infection, causes of which remain unknown^18–20^.

In 2004, Brinkmann et al. described neutrophil extracellular traps (NETs), which actively release their DNA into the extracellular space, as a completely new form of neutrophil defense. NETs bind to gram-positive and gram-negative bacteria to differentiate and eliminate virulence factors^21^. During sepsis, neutrophils and monocytes/macrophages phagocytose bacteria that have invaded blood vessels, and cytokines bind complementary to Toll-like receptors (TLRs) and other receptors on neutrophils, activating neutrophils. Subsequently, calcium ions accumulated in the endoplasmic reticulum of neutrophils are released into the cytoplasm. An increase in the concentration of calcium ions in the cytoplasm activates protein kinase C (PKC). Activated PKC induces the activation of nicotinamide adenine dinucleotide phosphate (NADPH) oxidase and ROS production^22,23^. ROS induces transfer of peptidylarginine deiminase 4 (PAD4) to the nucleus, where it changes arginine in the histone tails to citrulline, weakening its binding to DNA by changing its conformation, causing DNA to decondense and be released from the cell^24,25^. Neutrophils stimulated by high mobility group protein B1 (HMGB1) released via LPS or dead gram-negative rods mediated activation, release NETs composed of chromatin fibers containing DNA and citrullinated histones within 2–4 h. NETs include more than 30 proteins, including myeloperoxidase (MPO), neutrophil elastase (NE), and HMGB1^26,27^. Extracellular stimulators of NET production have been reported to be induced not only by LPS from gram-negative rods but also by substances such as PMA and monosodium urate crystals^28,29^.

Citrullinated histones, which are released from cells act as DAMPs^30^, disrupt the vascular endothelium in microvascular environments, thereby activating the blood coagulation system, and inducing septic DIC and deep vein thrombosis^31–33^. NETs that have accomplished their functions in vivo are degraded by DNase I in serum. However, in the blood of SLE patients, DNase I activity is reduced due to autoantibodies targeting DNA and histones, thereby interfering with NET clearance^20,34^.

In this study, we aimed to elucidate the cause of high presepsin levels in SLE and hemophagocytic syndrome and demonstrate a novel mechanism of presepsin production by monocytes and macrophages phagocytosing NETs, in addition to the known presepsin production mechanism.

## Results

### NETs are induced by bacterial stimulation in a concentration-dependent manner

**Figure 1:**
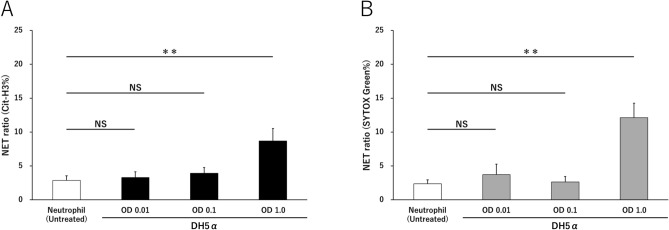
NETs increase in concentration-dependent manner after DH5α stimulation. NETs were induced in a concentration-dependent manner by bacterial (DH5α) stimulation. (**A**,**B**) DH5α cells were incubated with neutrophils for 4 h under platelet-free conditions, and Cit-H3 and nuclear released into the extracellular space were measured as NET ratio. **p < 0.001, when compared with untreated neutrophils. Data represent the mean values ± SD of at least three experiments. NS, not significant.

In the induction of NETs by DH5α stimulation of peripheral blood isolated neutrophils, both the ratios of Cit-H3 and SYTOX Green were not significantly different from untreated neutrophils at OD 0.01 and 0.1. However, at OD 1.0, the Cit-H3 NET ratio was 8.7 ± 1.8% (Untreated 2.9 ± 0.7%) and the SYTOX Green NET ratio was significantly increased to 12.1 ± 2.1% (Untreated 2.4 ± 0.6%) (*p* < 0.01) (Fig. 1A,B).

### NETs express high levels of extracellular CD14, MPO, and NE

**Figure 2:**
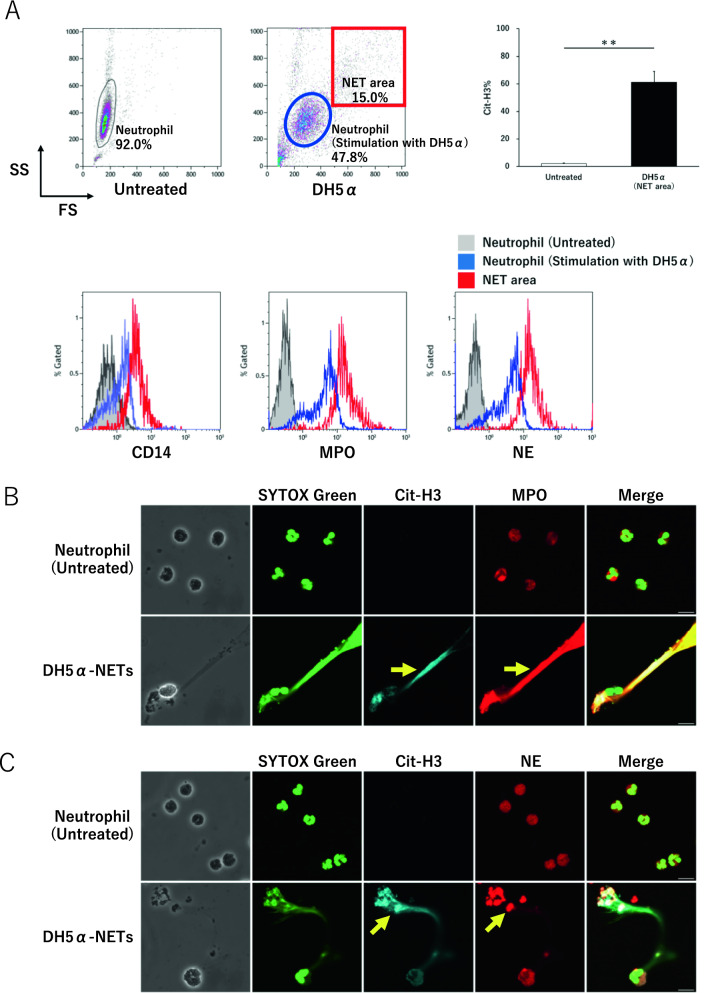

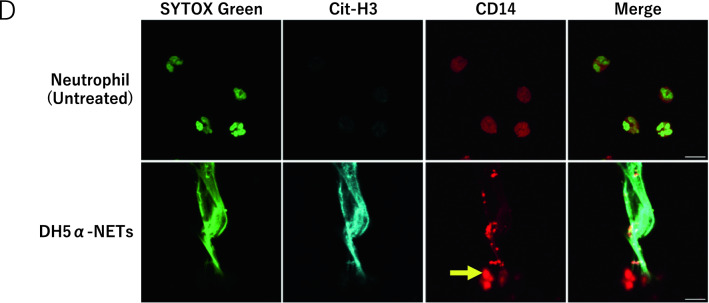
NETs stimulated with DH5α express high levels of CD14, MPO, and NE. (**A**) Cit-H3 positivity rates in Untreated and the NET area were measured by flow cytometry and compared. The NET area highly expressed CD14, MPO, and NE than the untreated neutrophils, as shown by flow cytometry. (**B**, **C**) Scale bar: 10 μm. Extracellular MPO and NE expression in DH5α-NETs by immunofluorescence imaging. (**D**) CD14 expression in DH5α-NETs. Scale bar: 10 μm.

The Median fluorescence intensity (MFI) values of CD14, MPO, and NE on the cell surface by flow cytometry were 0.7 ± 0.0%, 0.3 ± 0.0%, and 1.7 ± 0.5% for untreated neutrophils and 1.6 ± 0.2%, 2.3 ± 0.6%, 6.0 ± 0.6% for neutrophils stimulated by DH5α, while the NET area was 5.1 ± 0.9%, 13.3 ± 1.9%, 28.1 ± 3.4%, respectively. CD14, MPO, and NE were highly expressed in the NET area than in the untreated cells. Specifically, the expression of CD14, the source of presepsin, was significantly higher in the NET area than in the neutrophils (Fig. 2A).

### Evaluation of extracellular citrullinated histones and CD14 in NETs by immunofluorescence imaging

Although citrullinated Histone H3 (Cit-H3) was negative in untreated neutrophils, it was positive upon stimulation with DH5α, revealing the presence of NETs.

The extravasated nuclei of these cells stained positively with SYTOX green. The Cit-H3-positive NETs were extracellularly positive for MPO and NE (Fig. 2B,C). NETs stimulated by DH5α showed a higher CD14 expression than that in the untreated neutrophils, consistent with flow cytometry measurements (Fig. 2D).

### Evaluation of presepsin production in the induction of NETs

**Figure 3:**
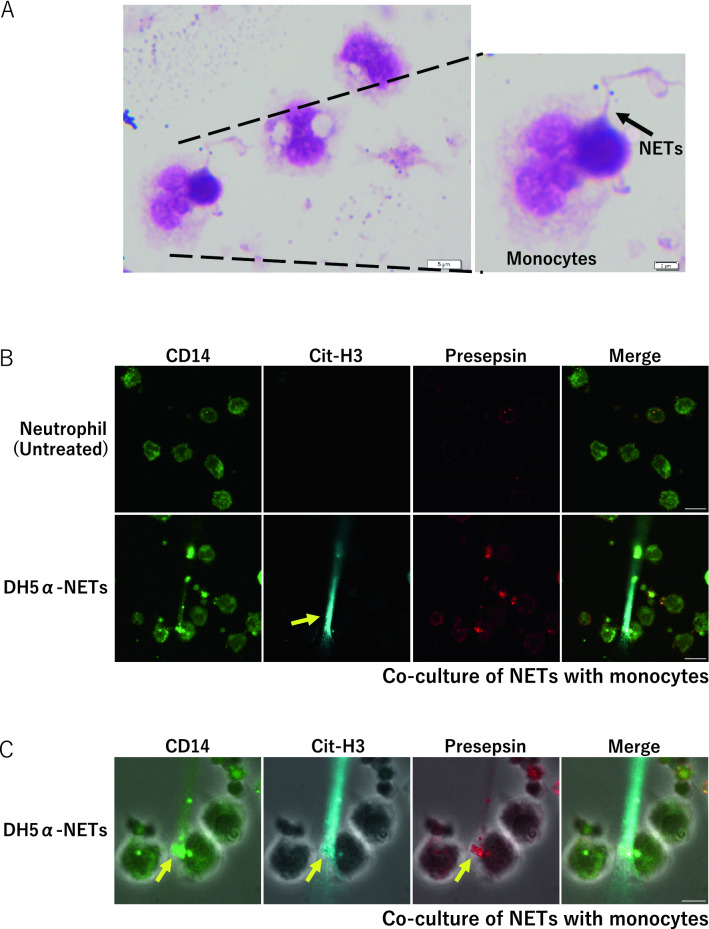

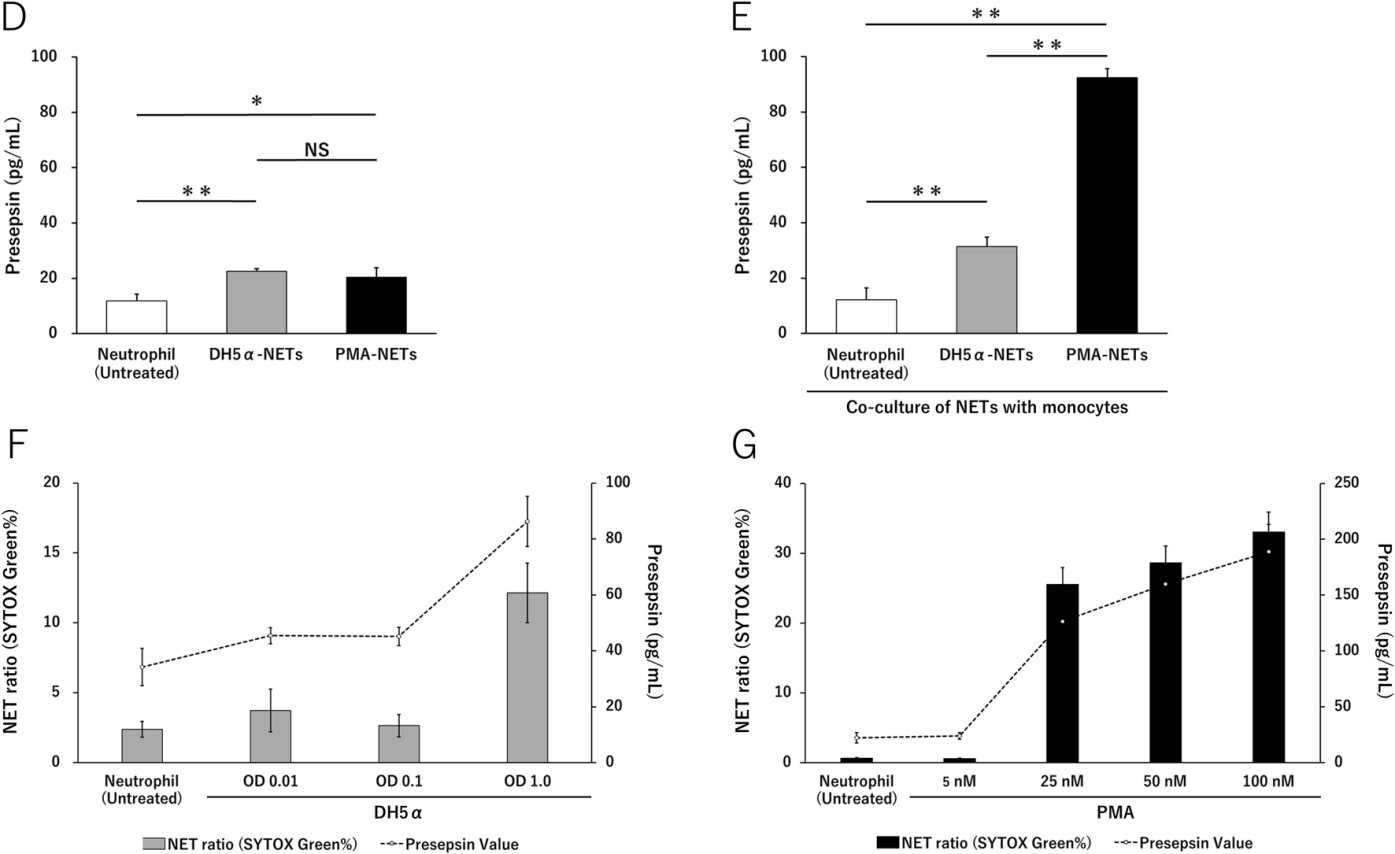
Presepsin levels increase as the monocytes phagocytose NETs. (**A**) A May–Giemsa-stained image 3 h after PMA-NETs were induced and co-cultured with monocytes. (1,000 × magnification). The scale bar is 5 μm, and the scale bar in the magnified image is 2 μm. (**B**) Immunofluorescence imaging of co-culture with monocytes after induction of DH5α-NETs. The arrow shows cells positive for citrullinated histones. Scale bar: 10 μm. (**C**) The arrow point indicates the area where monocytes are phagocytosing NETs. The scale bar in the magnified image is 5 μm. (**D**) Presepsin levels in DH5α (OD 1.0)-NET induction (NET ratio 14%), presepsin levels in PMA (50 nM)-NETs induction (NET ratio 23%). (**E**) Comparison of presepsin levels after phagocytosis by co-culturing with monocytes for 3 h. (**F**,**G**) Variation in NET ratio and presepsin values with DH5α and PMA concentrations are shown. *p < 0.05 and **p < 0.001, when compared with untreated neutrophils. Data represent the mean values ± SD of at least three experiments. NS, not significant.

After inducing PMA-NETs, we observed the morphological images of monocytes that phagocytosed NETs (Fig. 3A). NETs and monocytes were co-cultured and stained for CD14, citrullinated histones, and presepsin and evaluated by immunofluorescence imaging. Citrullinated histones were not observed in untreated cells, but DH5α-NETs were positive for the same (Fig. 3B). In addition, monocytes phagocytosed NETs with high CD14 expression, and presepsin was produced intracellularly in monocytes (Fig. 3C). Presepsin levels in untreated neutrophils, DH5α-NETs-induced NET supernatants were compared. Compared with presepsin levels in untreated neutrophils (11.8 ± 2.4 pg/mL), those in DH5α-NETs (22.5 ± 0.8 pg/mL) and PMA-NETs (20.4 ± 3.4 pg/mL) showed a statistically significant increase but did not show a dramatic difference (Fig. 3D). The induction of NETs alone did not result in significant changes in presepsin levels. However, when monocytes were co-cultured with NETs, presepsin levels in DH5α**-**NETs (31.4 ± 3.4 pg/mL) and PMA-NETs (92.4 ± 3.3 pg/mL) increased substantially (Fig. 3E). Furthermore, the NET ratio increased in a concentration-dependent manner under both DH5α and PMA stimulation. Presepsin levels increased similarly as monocytes phagocytosed NETs (Fig. 3F,G).

### Presepsin is produced by monocytes to phagocytose NETs

In both DH5α-NETs and PMA-NETs, extracellularly released Cit-H3 was observed, and CD14 was highly expressed. In addition, CD14 and presepsin were found in cells phagocytosed by monocytes (Fig. 3C). Monocytes phagocytose NETs and take them into the cells to produce presepsin.

### Presepsin levels are lowered by suppressing NETs

**Figure 4:**
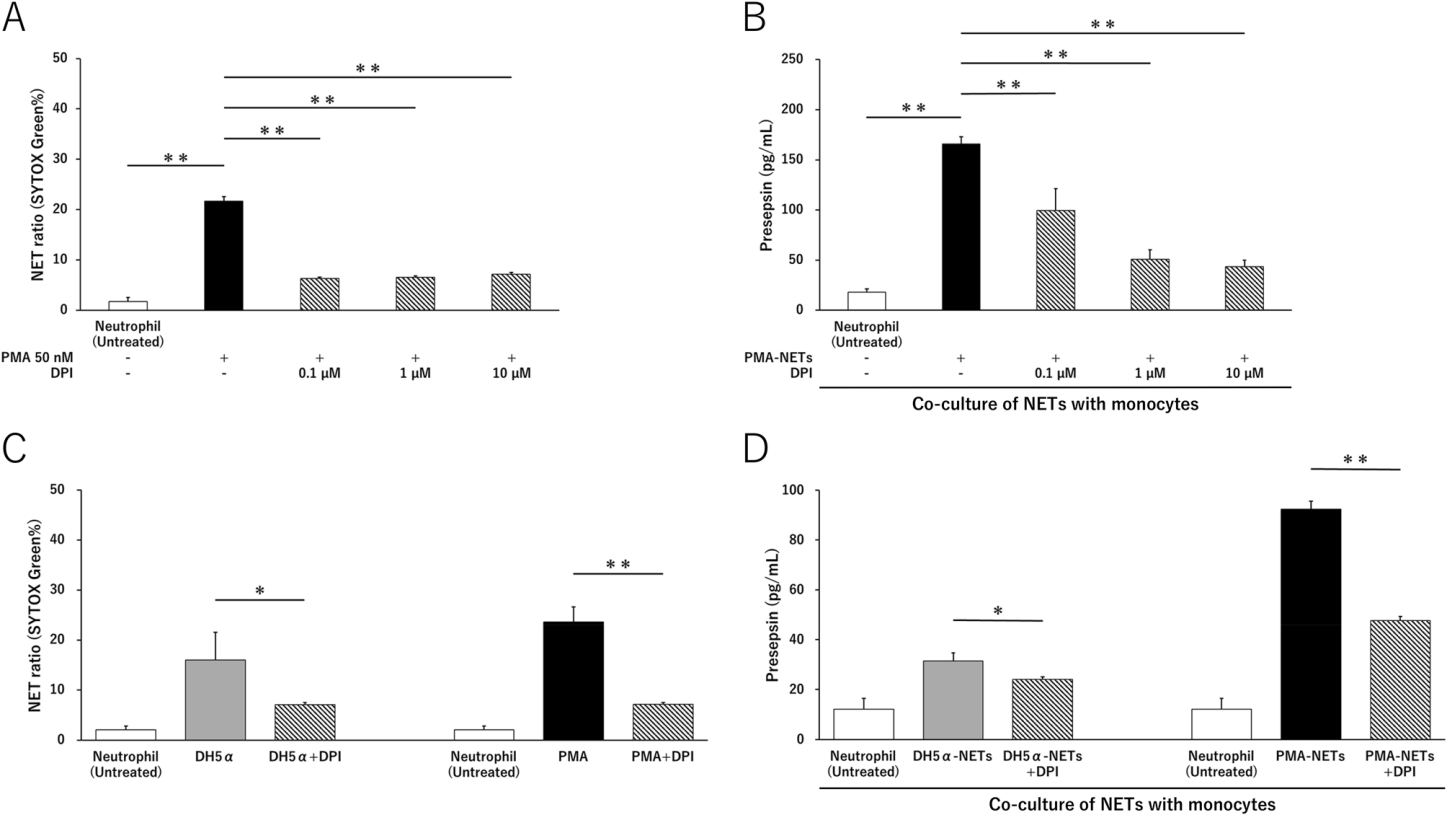
Presepsin levels decrease after NET suppression followed by co-culture with monocytes. (**A**) After neutrophils were treated with DPI for 30 min, the NET ratio in PMA stimulation was measured. (**B**) Comparison of presepsin levels after phagocytosis by co-culturing with monocytes for 3 h. (**C**) NET ratio by DH5α (OD 1.0) and PMA (50 nM) stimulation after DPI (10 µM) treatment. (**D**) After DH5α (OD 1.0)-NETs and PMA (50 nM)-NETs were suppressed by DPI (10 µM), presepsin levels were compared when the cells were co-cultured with monocytes for 3 h. *p < 0.05 and **p < 0.001, compared with untreated neutrophils. Data represent the mean values ± SD of at least three experiments.

We examined the effect of diphenyleneiodonium chloride (DPI) on presepsin production under conditions in which NETs were suppressed. Compared to that in PMA-NETs, the NET ratio in DPI (0.1–10 μM) pretreatment before PMA stimulation was lower, and NETs were suppressed (Fig. 4A). Furthermore, when the cells were co-cultured with monocytes and phagocytosed by monocytes, presepsin levels lowered in a DPI concentration-dependent manner. In other words, presepsin levels decreased in a concentration-dependent manner when NETs were suppressed (Fig. 4B). Inhibition of NETs by 10 μM DPI suppressed both DH5α-NETs and PMA-NETs. Similarly, the presepsin levels after co-culture with monocytes also decreased, and the presepsin level reflected NET ratio (Fig. 4C,D).

### Evaluation of presepsin for NET suppression using cytochalasin D and sivelestat

**Figure 5:**
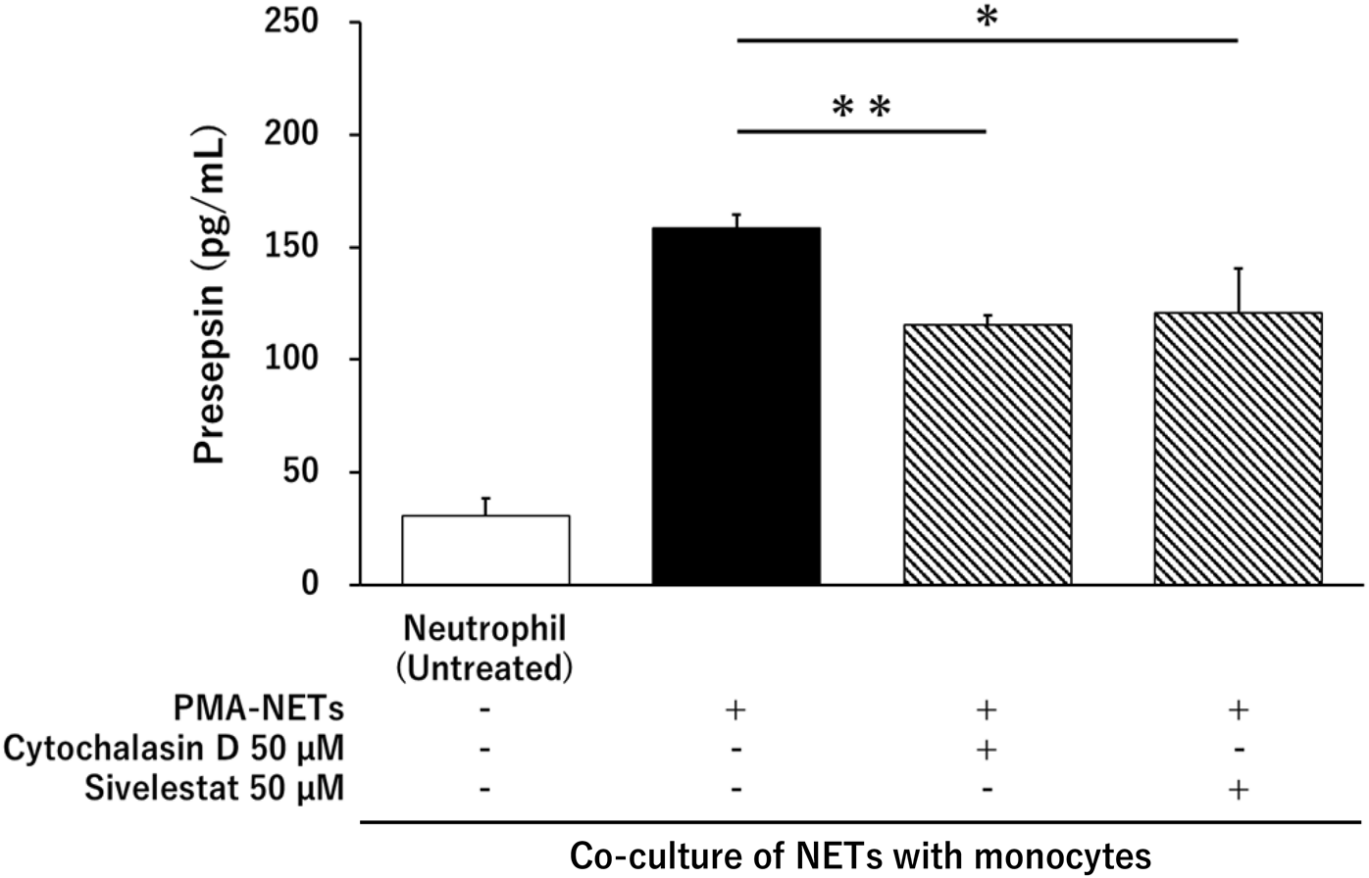
Inhibiting the phagocytic activity of monocytes decreases presepsin levels. Effect of phagocytosis and neutrophil elastase inhibitors on presepsin production by monocytes after induction of NETs with PMA (50 nM). *p < 0.05 and **p < 0.001, compared with untreated neutrophils. Data represent the mean values ± SD of at least three experiments.

Presepsin levels were evaluated under conditions where the phagocytic activity of monocytes was inhibited by cytochalasin D. In addition, we evaluated presepsin levels using sivelestat, an inhibitor of neutrophil elastase, which is a proteolytic enzyme of monocytes. Presepsin levels in neutrophils co-cultured with monocytes (untreated) were 30.7 ± 7.7 pg/mL; those in PMA-NETs co-cultured with monocytes were 158.7 ± 5.8 pg/mL; and those under conditions of cytochalasin D-inhibited phagocytosis were 115.7 ± 4.0 pg/mL. Inhibiting the phagocytic activity of monocytes significantly decreased presepsin levels (*p* < 0.01). In addition, under conditions of neutrophil elastase inhibition by sivelestat, the presepsin levels were 121 ± 19.5 pg/mL, which were significantly lower than those in the PMA-NETs phagocytosed by monocytes (*p* < 0.05) (Fig. 5).

### Presepsin is produced by macrophages to phagocytose NETs that express high CD14 levels

**Figure 6:**
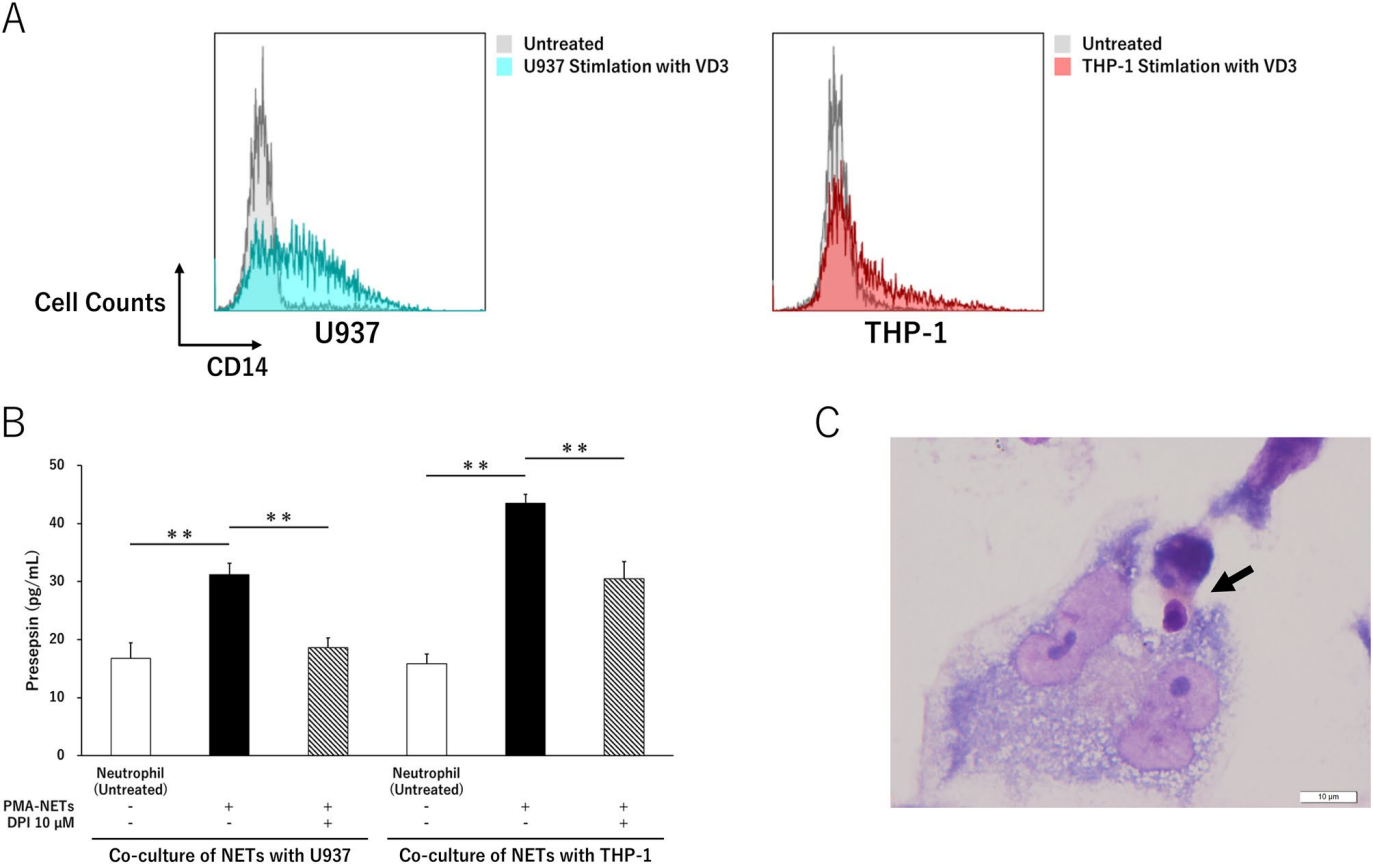
Presepsin levels increase as macrophages phagocytose NETs and decrease when NETs are suppressed. (**A**) Differentiation into macrophages used VD3 to induce CD14 expression against U937 and THP-1 cell lines. (**B**) Comparison of presepsin levels when co-cultured with PMA-NETs and macrophages. (**C**) Phagocytosis of PMA-NETs by THP-1 after macrophage induction visualized May–Giemsa staining. Scale bar: 10 μm. **p < 0.001, compared with untreated neutrophils. Data represent the mean values ± SD of at least three experiments.

CD14 expression in U937 and THP-1 cells showed mean fluorescence intensities (MFIs) of 1.3 and 1.5, respectively. Both cell lines showed significant CD14 expression with MFIs of 5.5 and 8.6 after 1α, 25-dihydroxyvitamin D3 (VD3) stimulation to induce differentiation into macrophages (Fig. 6A).

Presepsin levels (untreated) in VD3-stimulated U937 and THP-1 cells were 16.8 ± 2.6 and 15.9 ± 1.7 pg/mL, respectively, while those after co-culture of PMA-NETs with U937 and THP-1 cells increased to 31.2 ± 2.0 and 43.5 ± 1.6 pg/mL, respectively. In addition, the presepsin levels in both cell lines decreased after NETs were suppressed by DPI treatment and the cell lines were co-cultured (Fig. 6B). Morphological observation after 4 h of co-culture of the THP-1 cells and PMA-NETs, in which CD14 expression was induced by VD3 stimulation, showed that THP-1 cells phagocytosed NETs (Fig. 6C).

## Discussion

It has been reported that when neutrophils and monocytes phagocytose infectious bacteria in blood vessels, CD14 expressed on their cell surface is taken up by the cells, degraded by neutrophil elastase, and released as presepsin into the blood^14^. However, there are reports of high presepsin levels in patients with SLE without infection, and the cause of this has not been clarified^18,19^. We focused on NETs, which are known to increase in the blood in sepsis and in SLE patients, to elucidate the production mechanism of increased presepsin even in SLE patients who do not have an infection. Hakkim et al. reported that the number of NETs in the blood was higher in patients with SLE due to less degradation of NETs than in healthy subjects^20^. In addition to the conventional mechanism of presepsin production, we hypothesize a novel mechanism of presepsin production in which monocytes/macrophages produce presepsin by phagocytosing NETs.

Ratios of Cit-H3 and SYTOX Green in NETs that were released extracellularly from DH5α-stimulated neutrophils had increased in a concentration-dependent manner. However, in the case of NET induction, the NET ratio did not increase up to a DH5α culture OD of 0.1 but increased at an OD of 1.0, suggesting that a certain amount of infection is necessary for NET induction. In septic patients, when the amount of infectious bacteria in the blood is low, the bacteria are eliminated only by the phagocytic activity of neutrophils and monocytes; however, when the amount of infectious bacteria is high, the NET defense mechanism is activated in addition to phagocytic activity^35^. The induction of NETs by DH5α at an OD of 1.0 is thought to replicate the appearance of NETs in sepsi*s *in vitro.

NETs are formed in PMA-NETs by activating neutrophil protein kinase C (PKC) to activate NADPH oxidase^36^. NETs include suicidal NETs, which are ROS production-dependent, and vital NETs, which are non-ROS production-dependent. It has been reported that suicidal NETs release citrullinated histones into the extracellular space and die after the sterilization of bacteria^35,37^. Masuda et al. have described the fractions with high forward scatter (FS) and side scatter (SS) that appeared after stimulation with DH5α as the NET area^38–40^. In our experiments, post stimulation with DH5α, we also observed structures in the high FS and SS fractions that were not observed in unstimulated neutrophils (Untreated). We compared the expression levels of Cit-H3 in the structures in the high FS and SS fractions with those in unstimulated neutrophils (Untreated), and found that the structures in the high FS and SS fractions expressed significantly more Cit-H3, considering that NET formation was induced. In the NET area, CD14, MPO, and NE expressions were higher than that in DH5α-activated neutrophils, suggesting that cells in the NET area are highly capable of defending themselves by increasing the expressions CD14, which serves as a sensor for recognizing bacteria, and MPO and NE, which sterilize the bacteria.

MPO released extracellularly causes MPO cytoplasmic antibody (MPO-ANCA)-associated vasculitis, leading to a vicious cycle of inflammation^41–44^. Therefore, presepsin, which can be measured easily and rapidly, could be used as a biomarker for these diseases. In studies on septic patients and mouse models, it has been reported that CD14 expression increases in an MYD88-dependent manner^13^. In our experiments, CD14 expression in neutrophils increased after stimulation with DH5α. Interestingly, flow cytometry and immunofluorescence imaging revealed that CD14 expression was higher in the NET area Braian et al. reported that macrophages phagocytosed *Mycobacterium tuberculosis*-induced NETs^45^. Similarly, our immunofluorescence imaging using a presepsin antibody showed that monocytes phagocytosed NETs showing high CD14 levels in both DH5α- and PMA-stimulated NETs, demonstrating the production of presepsin in monocytes. However, compared to the isolated neutrophils, no significant change in presepsin levels was observed after induction of NETs by DH5α or PMA, suggesting that NETs do not produce presepsin; rather, monocytes/macrophages produce presepsin by phagocytosis of NETs. In particular, PMA-NETs only induced NETs in the absence of bacteria, which were phagocytosed by the monocytes, resulting in an increase in presepsin levels. These results indicate that monocytes phagocytose NETs that express high CD14 levels and produce presepsin in monocytes. In addition, when the NET number was increased in a PMA concentration-dependent manner and monocytes were phagocytosed, the presepsin level also increased as the NET ratio increased, demonstrating that the number of NETs had a significant effect on presepsin production. The induction of PMA-NETs was inhibited by DPI, an inhibitor of NADPH oxidase, and the presepsin levels decreased when the cells were co-cultured with monocytes. This result demonstrated that monocytes phagocytose NETs and thus produce presepsin and the number of NETs phagocytosed directly reflects the amount of presepsin produced under the same conditions in monocytes. Similar results were obtained for DH5α-NETs.

To prove that NETs released extracellularly by neutrophils were phagocytosed by monocytes in co-cultures , we performed a phagocytosis inhibition test using cytochalasin D, which inhibits the actin polymerization of phagocytes. Presepsin production was inhibited when NETs were co-cultured with monocytes. This may be because monocytes cannot phagocytose NETs. In addition, when sivelestat, an inhibitor of neutrophil elastase, was added before co-culturing NETs with monocytes, presepsin production was suppressed. These results demonstrated that neutrophil elastase intracellularly degrades CD14 expressed by NETs after monocytes phagocytose NETs and presepsin is produced by monocytes to phagocytose NETs.

We hypothesized that macrophages in tissues also phagocytose NETs and produce presepsin, as the stagnation time of monocytes in peripheral blood is several hours in vivo. Presepsin levels decreased after inhibition of PMA-NETs using DPI in PMA-NET co-culture with macrophage-induced cell lines, suggesting that macrophage-induced cell lines produce presepsin via phagocytosis of NETs. In particular, differentiated machrophage THP-1 cells actively phagocytosed PMA-NETs and showed increased presepsin levels relative to those in U937 cells. We concluded that monocytes and macrophage cell lines that phagocytose NETs were extracellularly released by neutrophils and produce presepsin.

**Figure 7:**
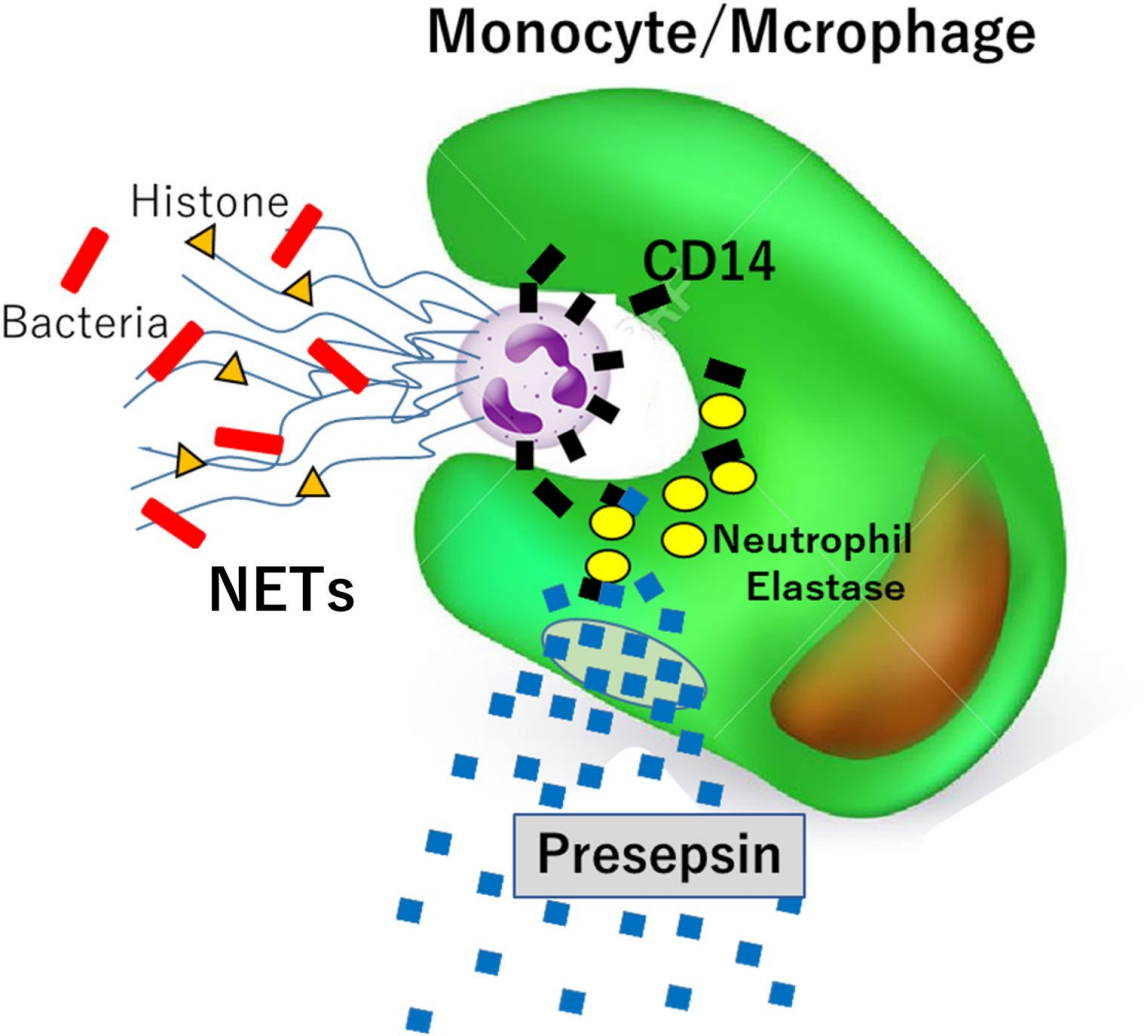
Schematic representation of the mechanism of presepsin production by monocytes/macrophages by NETphagocytosis. Neutrophils invoke NETs in response to infectious bacteria in blood vessels, trapping bacteria and turning them into dead cells. Phagocytosis of NETs by monocytes/macrophages results in intracellular degradation of CD14, which is expressed highly in NETs, and the production of a presepsin, which is released into the extracellular space.

Collectively, these results indicate that when the level of NETs increase in the blood owing to sepsis or SLE, monocytes/macrophages phagocytose NETs that express high CD14 levels on their cell surface. Thereafter, CD14 is degraded by neutrophil elastase in monocyte/macrophage cells to produce presepsin. Furthermore, in cases of increased presepsin levels in hemophagocytic syndrome, we speculated that phagocytes phagocytose CD14-expressing blood cells, resulting in increased presepsin levels (Fig. 7). The new mechanism of presepsin production is of great clinical significance because it not only explains the increased presepsin levels in SLE but also has the potential to be used as an indicator of SLE treatment efficacy. Further evidence on the mechanisms of the crosstalk between monocyte/macrophage and NETs will aid in the identification of novel therapeutic strategies for SLE and hemophagocytic syndrome.

## Methods

The content and execution of the current study were approved by The Ethical Committee of the Kagawa Prefectural University of Health Sciences, Japan (No.327). All methods were carried out in accordance with the guidelines and regulations of The Ethical Committee of the Kagawa Prefectural University of Health Sciences. Written informed consent was obtained from the participants before the study.

### Healthy donor samples and cell lines culture

The study was approved by the ethics committee of Kagawa Prefectural University of Health Sciences, and consent was obtained from the participating students and teaching staff (*n* = 7). U937 cells (American Type Culture Collection: CRL-1593.2) and THP-1 cells (American Type Culture Collection: TIB-202) were grown in RPMI1640 medium (Cat, No. R8758; Sigma-Aldrich, St. Louis, MO, USA) containing 10% (v/v) heat-inactivated fetal bovine serum (FBS) in a humidified atmosphere with 5% CO_2_ at 37 °C.

### Antibodies, proteins, and chemicals

The details of reagents used in this study and their sources are as follows: diphenyleneiodonium chloride (DPI; Cat, No.81050; Cayman Chemical Company, Ann Arbor, MI, USA), cytochalasin D (Cat, No.11330; Cayman Chemical Company), sivelestat (Cat, No.17779; Cayman Chemical Company), phorbol 12-myristate 13-acetate (PMA; Cat, No.P8139; Sigma-Aldrich), 1α, 25-dihydroxyvitamin D3 (VD3; Cat, No.71820; Cayman Chemical Company), May–Grünwald’s stain solution (Cat, No.15053; Mutokagaku, Tokyo, Japan), Gimsa’s stain solution (Cat, No.15002; Mutokagaku), 1/15 M phosphate buffer solution (pH 6.4) (Cat, No.15612; Mutokagaku), DAB Stain Kit ( Cat, No.15712; Mutokagaku), anti-human myeloperoxidase mouse monoclonal antibody (Cat, No.sc-52707; Santa Cruz Biotechnology, Dallas, TX, USA), anti-human neutrophil elastase mouse monoclonal antibody (Cat, No.sc-55549; Santa Cruz Biotechnology), anti-human citrullinated histone H3 rabbit polyclonal antibody (Cat, No.ab5103; Abcam, Cambridge, UK), anti-human presepsin mouse monoclonal antibody (F1106-13–3 antibody; Mochida Seiyaku, Tokyo, Japan), anti-human CD14 mouse monoclonal antibody (Cat, No.14–0149-82; Invitrogen, Carlsbad, CA, USA), FITC-conjugated anti-human CD14 mouse monoclonal antibody (Cat, No.6603511; Beckman Coulter, Brea, CA, USA), Alexa Flour 405-conjugated goat anti-rabbit IgG (Cat, No.ab175652, Invitrogen), Rhodamine (TRITC) -conjugated goat anti-mouse IgG (Cat, No.SA00007-1; Cosmobio, Tokyo, Japan), Rhodamine (TRITC) goat anti-rabbit IgG (Cosmobio, Cat, No.SA00007-2, Tokyo, Japan), and *E. Coli* DH5α Competent Cells (Cat, No.9057; Takara Bio Shiga, Japan).

### Isolation of neutrophils

Neutrophils were isolated at room temperature from the EDTA-anticoagulated peripheral blood of healthy volunteers by density gradient centrifugation using Polymorphprep (Cat, No.1114683; Axis-Shield, Dundee, Scotland). After centrifugation for 30 min at 500 × *g*, the lower cellular fraction containing neutrophils was collected, serum-free RPMI 1640 medium was added, and the neutrophils were washed by centrifugation for 10 min at 400 × *g*. Purified neutrophils were assayed on a Sysmex XS-800i hematology analyzer (Sysmex Corporation, Kobe, Japan), the purity of neutrophils was confirmed to be more than 98.0%.

### Isolation of monocytes from PBMCs

After density gradient centrifugation using Polymorphprep, the upper cell fraction containing peripheral blood mononuclear cells (PBMCs) was collected, serum-free RPMI 1640 medium was added, and the cells were washed twice by centrifugation for 10 min at 100 × *g* to remove platelets. Thereafter, monocytes were isolated from purified PBMCs using the EasySep Human Monocyte Isolation Kit (Cat. No. 19359; Stemcell Technologies, Vancouver, BC, Canada) according to the manufacturer’s instructions. Purified monocytes were assayed on a Sysmex XS-800i hematology analyzer, the purity of monocytes was confirmed to be more than 94.0%.

### Immobilization and optical density of *E. coli* DH5α competent cells

*Escherichia coli* DH5α competent cells (DH5α) were used after formaldehyde fixation. To standardize the amount of immobilized DH5α, the optical density (OD) was measured and adjusted. The number of DH5α bacteria at OD 1.0 was equivalent to 3.0 × 10^5^/µL, as determined from the colony assay. For the induction of NET formation, 100 µL of the bacterial solution was added to 900 µL of the neutrophil suspension.

### Induction of NETs

The purified neutrophils were resuspended in RPMI 1640 medium supplemented with 10% FBS. Purified neutrophils (1.8 × 10^6^ cells/well) were seeded in 24-well plates (Cat. No. SIAL0526; Sigma-Aldrich) and stimulated by a DH5α bacterial solution with an OD of 1.0, for 4 h at 37 °C (DH5α-NETs) or 50 nM PMA (PMA-NETs) for 2 h at 37 °C.

### Measuring the NET ratio

After induction of NETs, SYTOX Green was added to 100 µL of the cell suspension and incubated for 20 min at room temperature, and unwashed cells were analyzed using a flow cytometer (Cell Lab Quanta SC; Beckman Coulter). Similarly, anti-human citrullinated histone H3 rabbit polyclonal antibody was added to 100 μL of the cell suspension and incubated for 20 min at room temperature. Then, Rhodamine (TRITC) goat anti-rabbit IgG was added to the solution, incubated for 20 min at room temperature, and unwashed cells were analyzed using a flow cytometer. The positivity rates of Cit-H3 and SYTOX Green were evaluated as the NET ratio^38–40^.

### Comparison of neutrophils before and after DH5α stimulation using flow cytometry

The population of Cit-H3 positive cells in the NET area was determined to be more than 60% in the Side scatter and Forward scatter plots after DH5α stimulation. The expression levels of CD14, myeloperoxidase (MPO), and neutrophil elastase (NE) in neutrophils (untreated) and the NET area after 4 h of DH5α addition were compared by measuring the rate of fluorescence emitted from positive cells.

### Evaluation of Presepsin levels by co-culturing with monocytes after NET induction

PMA-NETs were collected by pipetting to make a neutrophil suspension containing NETs. Purified monocytes (5.0 × 10^5^ cells per well) from the peripheral blood of the same subject were seeded in 24-well plates, and the ratio of purified neutrophils (control) to that of the neutrophil suspension containing NETs added was the same. After incubation for 3 h at 37 °C, presepsin in the supernatant was measured using PATHFAST (LSI Medience Corporation, Tokyo, Japan).

### Immunofluorescence imaging of NETs

Purified neutrophils were seeded in 24-well plates with submerged coverslips at the bottom, and NETs were induced using DH5α. After stimulation, the medium was removed, and the remaining cells were washed with phosphate-buffered saline (PBS). Cells on the coverslips were fixed with 4% paraformaldehyde for 10 min at room temperature. After washing with PBS, cells were incubated in PBS containing 5% rat serum for 60 min to block non-specific antibody binding. The samples were then incubated for 60 min with the following primary antibodies: anti-human citrullinated histone H3 (Cit-H3) rabbit polyclonal antibody (dilution 1:1,000), anti-human MPO mouse monoclonal antibody (dilution 1:2,000) or anti-human NE mouse monoclonal antibody (dilution 1:2,000) or anti-human CD14 mouse monoclonal antibody (dilution 1:500). After washing in PBS, each primary antibody binding was visualized using secondary antibodies coupled to Alexa Fluor 405-conjugated goat anti-rabbit IgG (dilution 1:1,000) and rhodamine (TRITC)-conjugated goat anti-mouse IgG (dilution 1:2,000), and the samples were stained for DNA (Sytox Green; dilution 1:2,000). After incubation for 60 min, samples were washed with PBS and embedded in 80% glycerol. All the procedures were performed at room temperature^46,47^. Images were obtained using a confocal microscope (FLUOVIEW FV10i; Olympus Corporation, Tokyo, Japan).

### Immunofluorescence imaging of monocytes that had phagocytosed NETs

Purified monocytes were seeded in 24-well plates with coverslips submerged at the bottom and co-cultured with monocytes after induction of NET formation. After incubation for 3 h at 37 °C, the medium was removed, and the remaining cells were washed with PBS. Cells on the coverslips were fixed with 4% paraformaldehyde. After washing with PBS, samples were incubated in PBS containing 5% rat serum for 60 min. Thereafter, the samples were incubated for 60 min with the following primary antibodies: anti-human cit-H3 rabbit polyclonal antibody (dilution, 1:1,000) and anti-human presepsin mouse monoclonal antibody (dilution, 1:5,000). After washing in PBS, each primary antibody binding was visualized using secondary antibodies coupled to Alexa Fluor 405-conjugated goat anti-rabbit IgG (dilution 1:1,000) and rhodamine (TRITC)-conjugated goat anti-mouse IgG (dilution 1:2,000). Subsequently, the samples were stained for DNA (Sytox Green; dilution 1:2,000) or CD14 using FITC-conjugated anti-human CD14 mouse monoclonal antibody. After incubation for 60 min, samples were washed with PBS and embedded in 80% glycerol. All the procedures were performed at room temperature. Images were acquired using a confocal microscope (FLUOVIEW FV10i).

### NET ratio and presepsin evaluation after inhibitor treatment

Neutrophils were treated with DPI, which inhibits NADPH oxidase activity. Prior to DH5α or PMA stimulation, purified neutrophils (1.8 × 10^6^ cells/well) were exposed to 10 μM DPI for 30 min at 37 °C. After NET inhibition, the NET ratio was analyzed using flow cytometry, and presepsin levels were measured after monocyte phagocytosis. Cytochalasin D was used as a phagocytosis inhibitor, and sivelestat was used as an neutrophil elastase inhibitor. Before phagocytosis of NETs by monocytes, purified monocytes (5.0 × 10^5^ cells/well) were exposed to 50 μM cytochalasin D or 50 μM sivelestat for 30 min at 37 °C. After co-culture with NETs, the presepsin levels were measured.

### NET phagocytosis by macrophages increases presepsin levels

To differentiate U937 and THP-1 cells into active macrophage-like cells, the cells were resuspended in a culture medium containing 100 nM VD3 to a density of 2.0 × 10^5^ cells/mL and incubated for 48 h at 37 °C. After differentiation, the cells were co-cultured with PMA-NETs derived from neutrophils obtained from healthy volunteers for 3 h, and presepsin in the supernatant was measured.

### Statistical analyses

All statistical analyses were performed using SPSS version 24.0 (SPSS Inc, Chicago, IL, USA). The data are presented as the mean ± standard deviation (SD), and Student’s *t*-test was used for comparisons between two groups. A *p*-value of less than 0.05 was considered statistically significant. In the graphically represented data, *, and ** denote *p* values of less than 0.05, 0.01, respectively.

## Data Availability

On reasonable request to the corresponding author, data supporting the findings of this study will be available after approval from the Ethical Committee of the Kagawa Prefectural University of Health Sciences.
